# Metabolic enhancers: a new paradigm in cell culture media optimization

**DOI:** 10.1186/1753-6561-5-S8-P19

**Published:** 2011-11-22

**Authors:** Abi M Abitorabi, Christopher Wilcox

**Affiliations:** 1 GIRUS Life Sciences, Inc., Sunnyvale, CA, 94085, USA; 2 Sheffield BioScience, Beloit, WI, 53511, USA

## 

**Figure 1:**
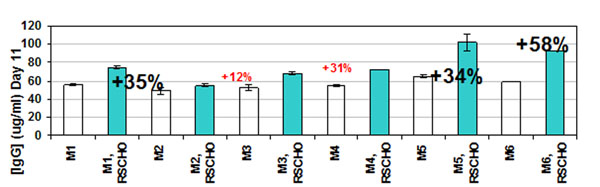
Antibody production by Chinese hamster ovary (CHO) cells increase with the Regocel™ small molecules (RS-CHO) in different commercial media (M1-M6).

Our primary focus is enabling and accelerating biological drug manufacture through the development of cost-effective technologies that facilitate rapid bioprocess development and improve manufacturing “bang-for-the-buck”. Using data mining and cell-based screens we have designed the Regocel™ small molecule yield enhancers for a number of mammalian cell platforms used in biomanufacturing such as CHO and NS0 cells. Chemically defined molecules were screened against several parameters: growth, viability and most importantly protein production. Formulations were developed that are defined, animal-free, non-nutritional and compatible with different media, nutritional supplements, culture formats and scales. Because they target ubiquitous cellular pathways such as apoptosis, cell cycle and protein synthesis, these formulations provide consistent results with a variety of clones and can shorten bioprocess times and cost. Our results with Regocel™ supplements demonstrated increased protein production in a variety of commercial media (Figure 1), increased yields of different proteins such as antibodies and mammalian target of rapamycin (mTOR), immediate yield enhancements (from passage 1), and persistence of yield enhancements over many passages with no permanent alterations to cells, as determined by return of productivity to control levels upon Regocel™ supplement removal from the medium. These new small molecule formulations provide a new means of improving cell culture outcome independently of mammalian cell clone, cell culture medium and process.

